# Isolation of a wide range of minerals from a thermally treated plant: *Equisetum arvense*, a Mare’s tale

**DOI:** 10.1007/s00775-015-1320-0

**Published:** 2016-01-13

**Authors:** Anna Sola-Rabada, Julia Rinck, David J. Belton, Annie K. Powell, Carole C. Perry

**Affiliations:** Interdisciplinary Biomedical Research Centre, Nottingham Trent University, Clifton Lane, Nottingham, NG11 8NS UK; Karlsruhe Institute of Technology, Institute of Inorganic Chemistry, Engesserstrasse 15, 76131 Karlsruhe, Germany; Karlsruhe Institute of Technology DFG-Center for Functional Nanostructures (CFN), Wolfgang-Gaede-Straße 1a, 76131 Karlsruhe, Germany

**Keywords:** Silica, *Equisetum arvense*, Wollastonite, Calcium sulphide, Vitrification

## Abstract

Silica is the second most abundant biomineral being exceeded in nature only by biogenic CaCO_3_. Many land plants (such as rice, cereals, cucumber, etc.) deposit silica in significant amounts to reinforce their tissues and as a systematic response to pathogen attack. One of the most ancient species of living vascular plants, *Equisetum arvense* is also able to take up and accumulate silica in all parts of the plant. Numerous methods have been developed for elimination of the organic material and/or metal ions present in plant material to isolate biogenic silica. However, depending on the chemical and/or physical treatment applied to branch or stem from *Equisetum arvense;* other mineral forms such glass-type materials (i.e. CaSiO_3_), salts (i.e. KCl) or luminescent materials can also be isolated from the plant material. In the current contribution, we show the chemical and/or thermal routes that lead to the formation of a number of different mineral types in addition to biogenic silica.

**Electronic supplementary material:**

The online version of this article (doi:10.1007/s00775-015-1320-0) contains supplementary material, which is available to authorized users.

## Introduction

Silica is one of the most common materials in nature and is the second most common biogenic mineral after carbonates [1], and it is deposited in living organisms, including animals, plants and diatoms [2]. Horsetail (*Equisteum* spp.) is classified as one of the most ancient species of living vascular plants [3]. A remarkable characteristic of *Equisetum* species is their ability to take up and accumulate silica in their tissues giving the epidermis a rough texture [4]. Back in the 1980s, Williams and co-workers were one of the first groups to characterize the nature of silica in biological systems by using transmission electron microscopy, FTIR and NMR spectroscopy [5]. This natural silica, often referred to as biogenic silica, is present in the form of amorphous silica [5, 6] and for some plants seems to be an essential mineral for growth [7]. The ability of plants to produce biogenic silica with a wide range of morphologies under mild physiological conditions is of great interest to scientists (and industry); as it gives the material exceptional properties, such as ordered hierarchical porous structures applicable for catalysis [8], biosensing [9] and biomedical applications [9]. Further, plants and other biological organisms (i.e. sponges or diatoms) produce silica in amounts of gigatons per annum, whereas industrial processes only produce mere megatons. Biogenic silica in plants is present together with the organic matrix, including polymers (i.e. cellulose), proteins, other carbohydrates, lipids, metal ions (such as Ca, K, S, Cl, Na, Al and P), and phenolic compounds, which also play an important role in the hierarchical structures of biosilica [10]. The distribution and amount of silicon and other metal ions vary between individual plant families [11] and, within the plant, depending on the anatomical region studied [6]. For example, macrohairs from the lemma of the grass *Phalaris canariensis* L., showed a higher deposition of silicon, as silica throughout the macrohair for the mature stages of the plant; whereas for early stages (immature plant), silicon, as silica was mainly deposited at the tip of the microhair [6]. In addition, the silica formed in these plant hairs was remarkably free of metal ions [6]. In *Equisetum arvense*, silica was found to be located within the secondary cell wall providing mechanical strength and rigidity to the plant [4].

Biogenic amorphous silica from plants has been extracted by a number of different methods [12–17]. However, during the extraction procedure, there are also reports of the presence of crystalline silica forms, such as α-quartz, after removal of the cell wall matrix by ashing [18, 19]. Besides this, the presence of either other minerals (i.e. Ca^2+^ mineral types) [20] or metal ions in plant materials subjected to chemical and/or physical treatments, can result in the formation of new materials. As such, it is important to know both the composition of the native material and the effect of specific treatment(s) on the composition of the materials that may arise.

**Fig. 1:**
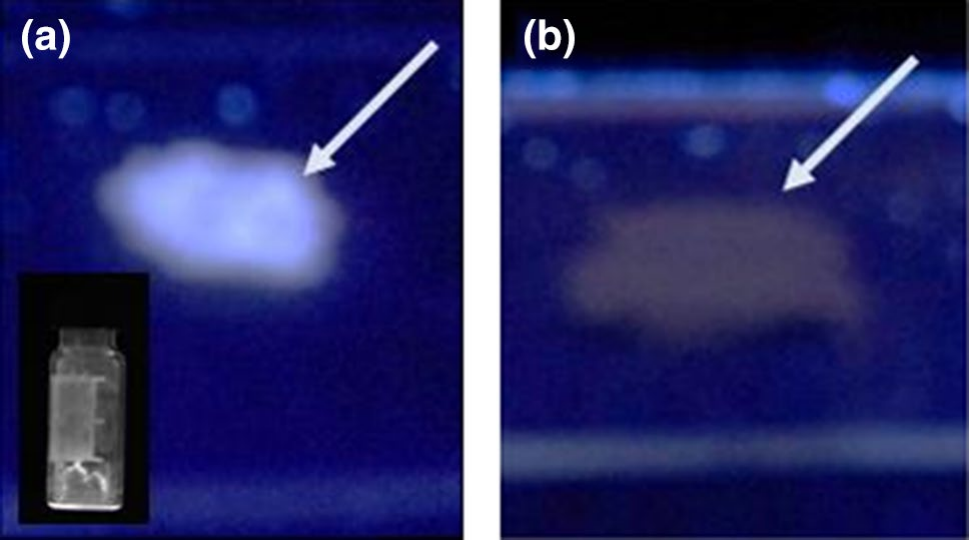
Samples (from the branches of *Equisetum arvense*) irradiated with *blue light* are **a** acetone treated/incomplete digestion/heat treated under H_2_/Ar and **b** non-acetone treated/incomplete digestion/heat treated under H_2_/Ar

The present study arose following two observations: (1) thermal treatment of samples of *Equisetum arvense*, using a conventional ashing approach yielded X-ray diffraction patterns that contained numerous peaks that were not related to silica and (2) following thermal treatment, samples occasionally showed luminescence behavior in the presence of UV light (Fig. 1). The study arose out of a need to understand where the luminescence comes from and was extended to include the effect of processing conditions on minerals formed from different parts of a plant, in this case the branches and stems of *Equisetum arvense*.

## Materials and methods

**Scheme 1:**
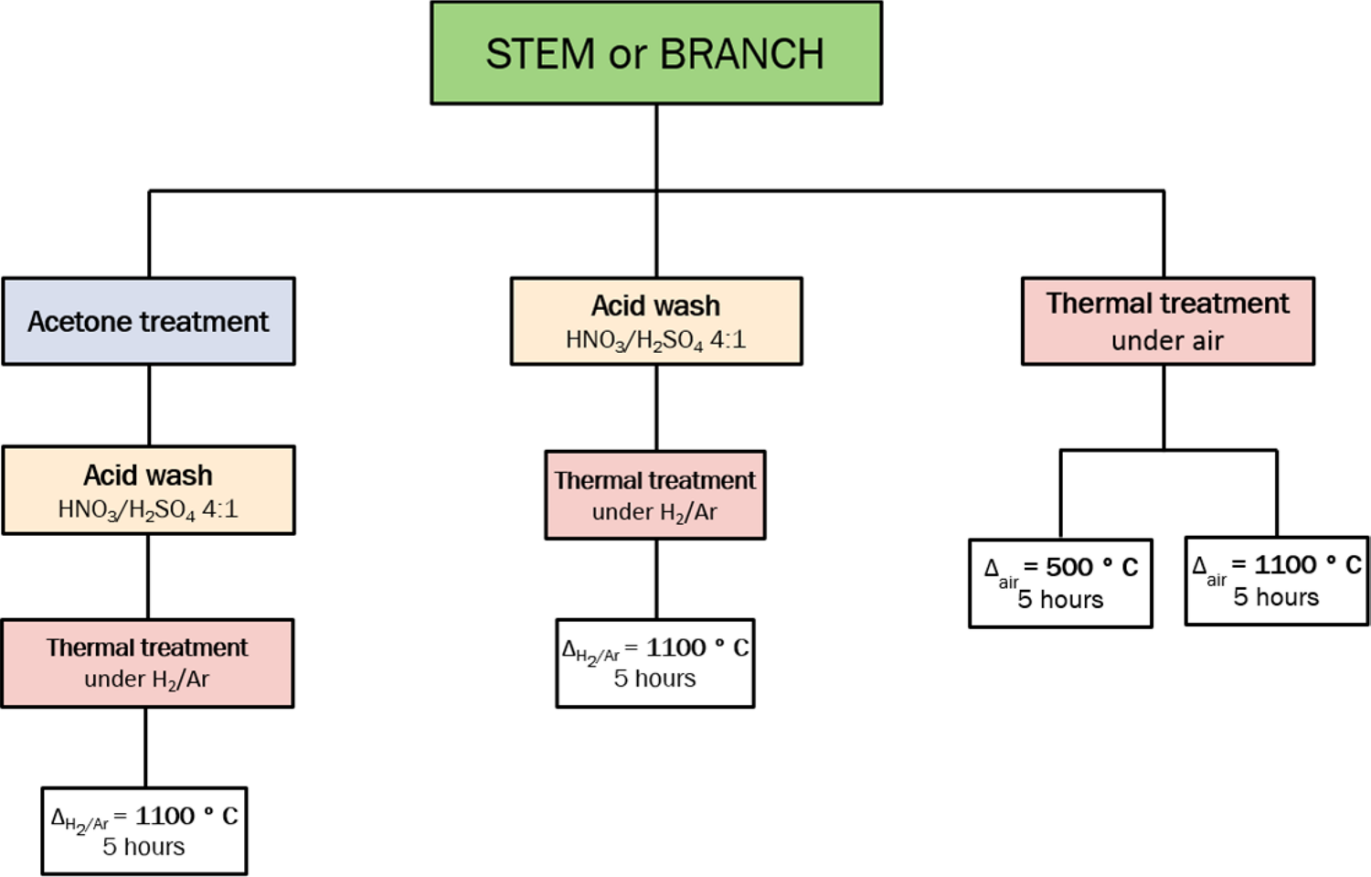
Methodology followed for the treatment of *Equisetum arvense* plant material

Stems and branches of *Equisetum arvense* were collected from the campus of Karlsruhe Institute of Technology, in Germany in May/June 2012. Fresh samples were dried immediately then stored at room temperature in the dark. A number of different treatments (Scheme 1) were performed to study the elemental composition, characteristics and properties of the materials generated. Plants were separated into stems and branches and dried in an oven at 50 °C prior to acid digestion in concentrated HNO_3_/H_2_SO_4_ (4:1). To avoid acid burning, the plant material was first covered with nitric acid and the sulphuric acid then added carefully into the system. The mixture was stirred and left in a fume hood until a fine white precipitate was obtained and the release of oxides of nitrogen had stopped, usually around 48 h. Precipitates were isolated and washed with copious amounts of deionized water until the pH value of the water was ca. 5. The samples were then lyophilized before being heat-treated either under H_2_/Ar or air at 1100 °C for 5 h with a heating rate of 10 °C/min up to the maximum set temperature. Another set of samples was prepared as above but, in addition, a treatment with acetone to remove chlorophyll was performed prior to the acid digest. After treatment, the acetone solution was decanted and any remaining solvent evaporated with the aid of a rotary evaporator. Finally, a comparison study was carried out where the native plant material was only heat treated (no acid digest) in air at temperatures of 500 and 1100 °C for 5 h with a heating rate of 10 °C/min up to the maximum temperature.

The morphology of the samples was determined by SEM (JEOL JSM-840A, 20 kV). Samples were attached to aluminum stubs using double-sided carbon adhesive tape and carbon coated (Edwards, sputter coater S150B). The crystallinity of the precipitates obtained was characterized using XRD (PANalytical X’Pert PRO, Cu Kα radiation with wavelength of 1.54056 Å). Ground samples (if necessary) were packed into an aluminum sample holder and scanned from 5° to 80° of 2*θ* at an accelerating voltage of 45 kV, 40 mA filament current, using a scan speed of 0.02° s^−1^ at room temperature. Diffraction patterns were analyzed using X’Pert-HighScore Plus (Version 2.0a) program for diffractogram manipulation, background determination and peak identification. For small amounts of sample, a polypropylene (PP) mask was fitted into the aluminum holder. ATR (Perkin Elmer Spectrum 100 FTIR Spectrometer with Diamond/KRS-5 crystal) was used to detect the functional groups present in the samples. Spectra were averaged from 32 scans at 4 cm^−1^ resolution with air as background. The organic content in the samples was determined by TGA (Mettler Toledo TGA/SDTA 851^e^). Samples were heated at 10° min^−1^ from 30 to 900 °C in air to ensure complete combustion of all organic material. The concentration of silicon (Si), calcium (Ca), potassium (K) and sulphur (S) in the samples was determined by using ICP-OES (Optima 2100DV). Samples (50 mg) were fused into a Ni crucible with solid ground NaOH (5 g) at 400 °C during 15 min using a temperature ramp of 10° min^−1^. Collected samples were dissolved in distilled water and after 1 h acidified with concentrated nitric acid (HNO_3_). Final samples (1000 ppm in 10 % HNO_3_) were filtered with a 0.45 μm disc filter prior to analysis. Standard solutions for each element were prepared from 1000 ppm stock solutions purchased from BDH Laboratory Supplies. The amount of each was determined by comparison with standards of known concentrations (0–100 ppm, correlation coefficient of 0.999) using a wavelength of 251.6 for Si, 317.9 for Ca, 766.5 for K and 182.0 for S. For measurement of luminescence, samples were irradiated with blue light (302 nm) and luminescence measured visually.

## Results and discussion

### Composition of the native plant material

**Fig. 2:**
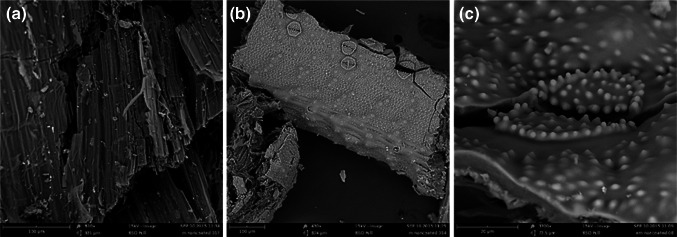
SEM images of *Equisetum arvense* plant showing two distinct types of structure: **a** multi-laminate layers, **b** *spotted* surface and **c** stomata shape within the *spotted* surface. *Scale bars* are 100 μm (**a**, **b**) and 20 μm (**c**)

Native *Equisetum arvense* was studied to identify the elemental composition and the minerals that could be present in the samples (branch and stem). SEM analysis (Fig. 2) showed, predominantly, two types of structures: multi-laminate layers (Fig. 2a) and spotted surfaces (Fig. 2b) which generally contained a stomatal shape (Fig. 2c), similar to structures identified previously [4].

**Fig. 3:**
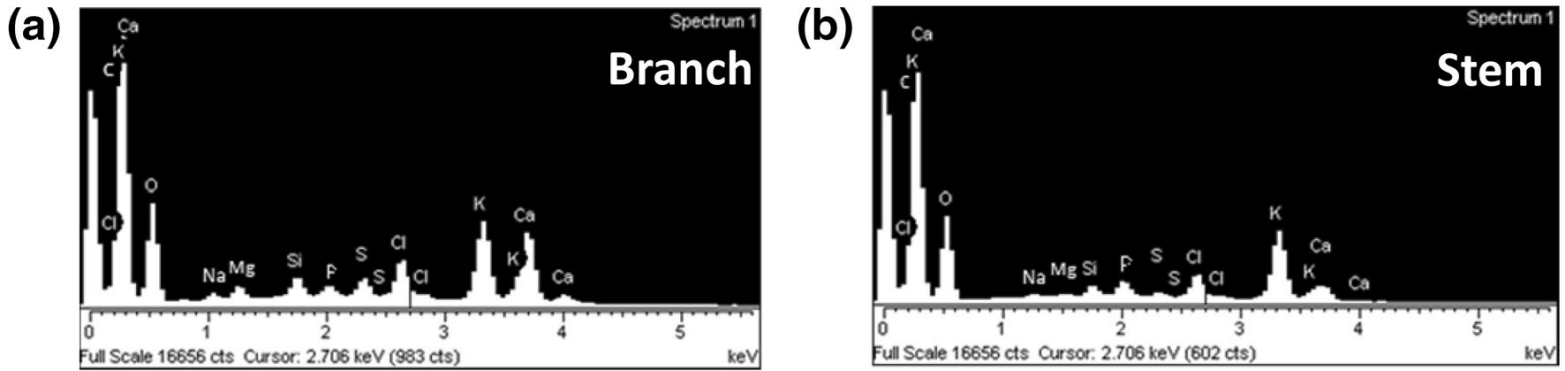
EDX spectra of *Equisetum arvense* **a** branch and **b** stem. All elements detected in the sample are labeled

EDX analysis (Fig. 3) showed the presence of a wide range of elements in the plant material. The atomic % of Si was found to be higher in the stem compared to the branch. These results were in accordance with the data obtained by ICP-OES where % of Si was 2.4 ± 0.1 % in branches and 3.8 ± 0.6 % in stems. Other elements, such as calcium (Ca), potassium (K), sulphur (S), sodium (Na), magnesium (Mg), chlorine (Cl) and phosphorus (P) were also detected in the samples (Fig. 3). Ca was more likely to be found in branches (2.6 ± 0.2 %) than in stems (1.1 ± 0.2 %); together with Mg, Cl, S and P (listed in order of decreasing %). In contrast, stems contained a higher amount of K compared with the branches, being 10.2 ± 0.5 % and 3.6 ± 0.2 %, respectively. *Equisetum* spp. has been used as a decontaminant of soils (known as phytoremediation) due to its potential for metal uptake from soil [21, 22]. Thus the elemental composition may vary depending on the geographical area and the conditions (contaminated or not) of the soil. The high concentration of potassium found may be due to their growth in a potassium-rich soil. Potassium in its ionic form (K^+^) is an essential macronutrient in plants as it is vital for many functions such as maintenance of electrical potential gradients across the cell membranes [23] or enzyme activation [24] and its uptake in plants can vary from 1 to 10 % in dry matter [25]. In contrast, the amount of silicon found in horsetail (dry matter: 2.5–4.8 %), was not influenced by the increase of silicon levels in the soil [16]. Sample mapping (Supplementary Figure 1) showed that structures with spotted surface contained mainly Si and O, whereas for the multi-laminate layers a higher amount of K and Ca was detected compared to the Si levels. Other elements were also more likely to be found in the multi-laminate structure. Previous studies have shown that the distribution and amount of silicon and other metal ions vary within the plant, depending on the anatomical region studied [6], being the mineralized silica more dense on the outer layer of the *Equisetum arvense* plant [26], which corresponds to the spotted surface shown in Fig. 2b–c. On the other hand, potassium can be found in the vacuole and the cytosol pools of the plant cells [27], whereas calcium is commonly found in the plant walls [28]. Note that, deposition of silica in plants has also been investigated using optical techniques by applying staining procedures based on the reactivity of silanol groups in biosilica such as methyl red, crystal violet lactone and silver amine chromate [29, 30]. And most recently, the fluorophore PDMPO has been shown to be an ideal fluorescent tracer for Si [31, 32].

**Fig. 4:**
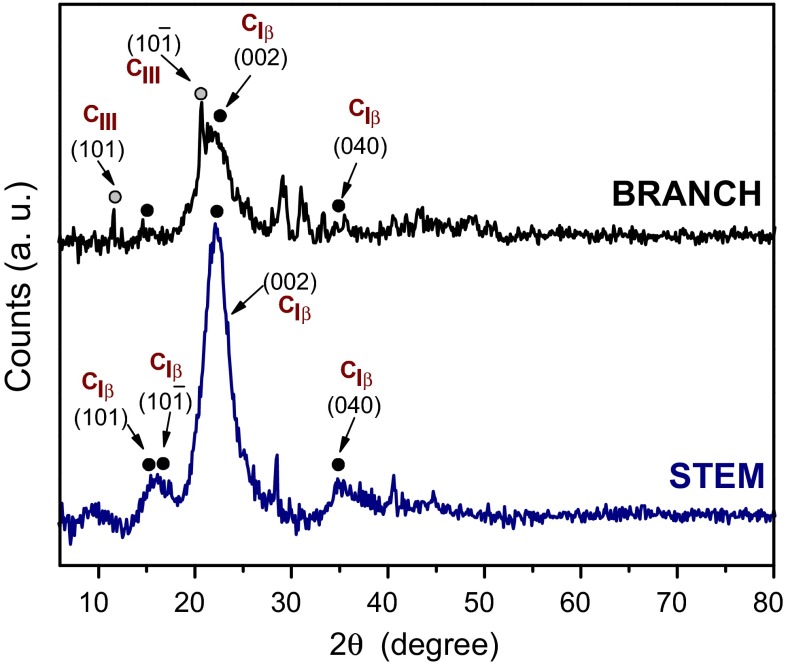
XRD diffractograms from native *Equisetum arvense* (branch and stem). Crystallographic phases identified are: cellulose I_β_ (*filled black circle*) and cellulose III_I_ (*filled gray circle*). Miller indices correspond to the crystal planes of cellulose I (C_I_) and cellulose III_I_ (C_III_)

XRD analysis of the native plant samples revealed typical reflections of cellulose (Fig. 4). Cellulose can exist in many polymorphs (Cellulose I, II, III and IV with additional sub-classifications) due to the hydroxyl groups within the structure being involved either in intra- or intermolecular hydrogen bonding which results in a different arrangement of atoms throughout the structure [33]. Both samples contained cellulose I_β_ (monoclinic) with a primary peak at 2*θ* ~22.5° corresponding to the (002) plane; and, a secondary broad peak (2*θ* ~16.0°) corresponding to the overlap of the (101) and (10) planes [34, 35]. The reflection at 2*θ* ~34.7° was attributed to the (040) plane. In the branches a number of the reflections not present in the diffractogram from the stem are suggested to arise from cellulose III even though this is not commonly found in plant materials being usually generated by treating cellulose I or II in liquid ammonia [36]. The peaks at ~11.6° and ~20.7° could be attributed to typical reflections of Cellulose III_I_ [37]. The peak situated at lower 2*θ* is attributed to the (101) planes, whereas the second peak is a contribution of the (10) and the (002) planes. Due to a higher order in the cellulose III structure [38] these reflections are expected to present a sharper appearance as is observed in this sample. The other two peaks found at ~29.1° and ~31.2° in the branches are at ~28.4° and ~40.6° in the stems could not be uniquely identified. Further, amorphous silica with a characteristic XRD peak around 2*θ* ~22.5° could not be discounted due to possible overlap with the (002) plane of cellulose I_β_ type.

**Fig. 5:**
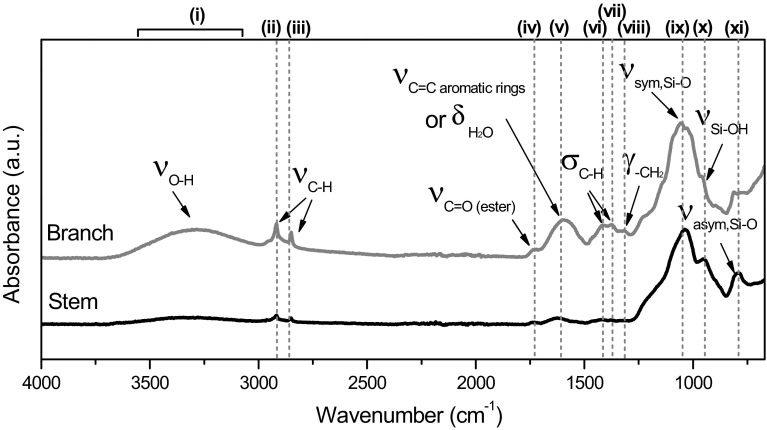
Peaks assigned from ATR spectra of native *Equisetum arvense* for the branches and the stems

ATR spectra of the samples (Fig. 5) presented a broad peak at 3600–3000 cm^−1^ (peak i) attributed to the O–H stretching mode in water (3500–3000 cm^−1^) [39] and/or hydrogen bonded silanol groups (3400–3200 cm^−1^) [40]. Bands between 3000 and 2800 cm^−1^ can arise from vibrations involving methyl and methylene cellulose groups [41]. Thus, the peaks at ~2918/2850 cm^−1^ (peaks ii and iii) and at ~1415/1376 cm^−1^ (peaks vi and vii) were attributed to C–H stretching and C–H bending modes, respectively [39]. The peak at 1318 cm^−1^ (peak viii) was also assigned to alkyl groups, specifically to the –CH_2_ wagging mode [39]. A stronger signal from the alkyl groups was observed in the branches than in the stems. These peaks can be associated with the presence of cellulose, lignin and hemicellulose present in the plants, as well as the presence of other substance such as waxes, essential oils or resins. Some of these compounds (i.e. waxes) may also show the CO vibration mode, which could be indicated by the peak detected at ~1740 cm^−1^ (peak iv) [42]. A broad peak at ~1628 cm^−1^ (peak v) can be either associated with adsorbed water in cellulose and/or hemicellulose [41, 43] or aromatic and C=C functional groups originating from waxes, fatty acids, fatty esters, or high molecular mass aldehydes or ketones [42]. Characteristic silica peaks at 1038 cm^−1^ (peak ix), 957 cm^−1^ (peak x) and 796 cm^−1^ (peak xi), corresponding to the antisymmetric Si–O–Si stretching mode, Si–OH stretching mode and symmetric Si–O–Si stretching mode, respectively [40], were detected.

**Fig. 6:**
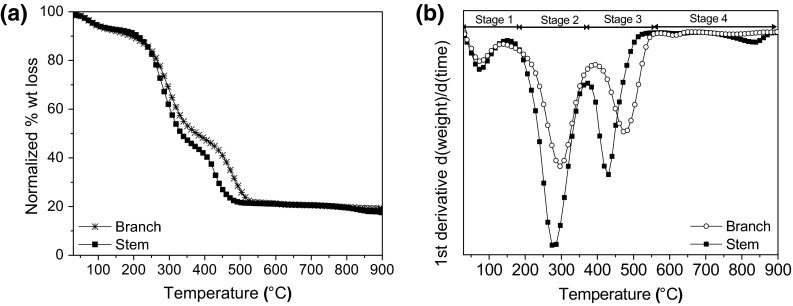
Thermal decomposition of native *Equisetum arvense* is shown as **a** TGA curves and **b** 1st derivative curve

Thermal decomposition (TGA analysis) of the plant material showed a slightly bigger total % weight loss (%wt) for the stems than the branches, being 81.8 ± 0.1 % and 80.4 ± 0.1 %, respectively. Four stages of weight loss were observed (Fig. 6). In the first stage, release of physisorbed water or volatile oils present in the native material (*T* < 120 °C) occurs. In the second stage (180–380 °C), the degradation of hemicellulose and cellulose pyrolysis gives the maximum rate of weight loss at ~278 °C for the stems and 294 °C for the branches [44]. For stage three, the maximum rate of weight loss was at ~477 °C and ~431 °C in the branch and stem, respectively. Full degradation of lignin has been reported, according to Wiedemann, between 380 and 450 °C [45]; or at even higher temperatures (400–800 °C), as reported by Lv et al.; but in all cases, the destruction of lignin occurs at a higher temperature than cellulose, due to the presence of aromatic compounds which makes lignin more thermally stable during pyrolysis [46]. The presence of lignin has been reported in vascular plants such as *Equisetum arvense* and the antiquity of this plant species corresponds with the appearance of lignin digesting enzymes in fungi [47]. Its content is typically less than cellulose [48] as shown in Supplementary Figure 2. Further, due to the complex structure of lignin, thermal stability can vary between types and species of plants [49], and, likewise (as observed in this study), within parts of the same plant. Plants that accumulate higher amounts of biogenic silica tend to present lower concentrations of lignin, as it is suggested that the presence of silica may substitute the mechanical role of lignin [50]. The last stage of weight loss (800–900 °C), was only observed in the stem. A higher amount of silicon (ICP-OES data), as well as, the presence of silanol groups was more apparent in stems than in the branches (ATR data); suggesting that the weight loss, in this case, can be associated to dehydroxylation of silanols. Different types of silanol groups can be present in silica: internal, vicinal, isolated and geminal, and, it has been reported that geminal silanols are removed from the surface at temperatures between 800 and 900 °C [51].

### The effect of thermal and/or chemical treatment on composition

#### Heat treatment in air

**Fig. 7:**
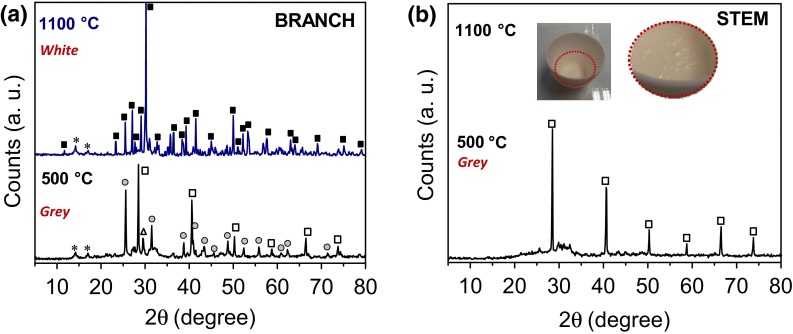
XRD diffractograms of *Equisetum arvense* after heat treatment under air for **a** branch and **b** stem. Crystallographic phases identified were: KCl (*open square*); CaSO_4_ (*filled gray circle*); C–S–H (*open triangle*) and CaSiO_3_ (*filled square*). Peaks arising from the PP holder are labeled with an *asterisk*. Sample color after treatment is indicated in *red*

Samples studied after heat treatment presented different crystalline composition (Fig. 7). Both, stems and branches, heated at 500 °C showed characteristic XRD diffraction peaks of KCl, corresponding to the cubic structure (JCPDS card No. 41-1476, space group Fm-3 m). Peaks at 2*θ* ~28.5°, 40.6°, 50.3°, 58.6°, 66.4° and 73.7° matched the (200), (220), (222), (400), (420) and (422) planes of KCl, respectively [52]. The presence of CaSO_4_, with an orthorhombic structure (JCPDS card No. 37-1496, space group *Amma*) was, however, detected in the branches. After heat treatment, amorphous silica was not detected in the branches, suggesting that the silica present (though detected by ATR analysis of native material) could have been transformed to a crystalline phase, either as crystalline silica or in combination with other elements. The peak at 2*θ* ~29.7° with a *d*-spacing value of 3.0 Å could be associated to (110) planes of calcium silicate hydrate (C–S–H) [53–55] and no pure crystalline silica was formed by heating (peaks expected for cristobalite, tridymite and quartz were all absent from XRD diffraction pattern). At higher temperature (1100 °C), the main crystalline phase observed was calcium silicate (CaSiO_3_) corresponding to a wollastonite like structure (JCPDS card No. 43-1460, space group P), which corroborated the assignment of the C–S–H peak in the sample heated at lower temperature. In contrast, the stems vitrified into the crucible on thermal treatment. The formation of glass from amorphous silica is typically performed in the presence of feldspar which contains the presence of alkaline fluxing agents (such as K, Ca and Na) [56], in this case, the presence of KCl, may have favored the reduction of the glass transition temperature (*T*_g_) for the formation of the glass. The XRD pattern of the vitrified sample containing Si, K and O only (Si:K ~3) is presented in Supplementary Figure 3. But it was not possible to identify the phases(s) present.

**Fig. 8:**
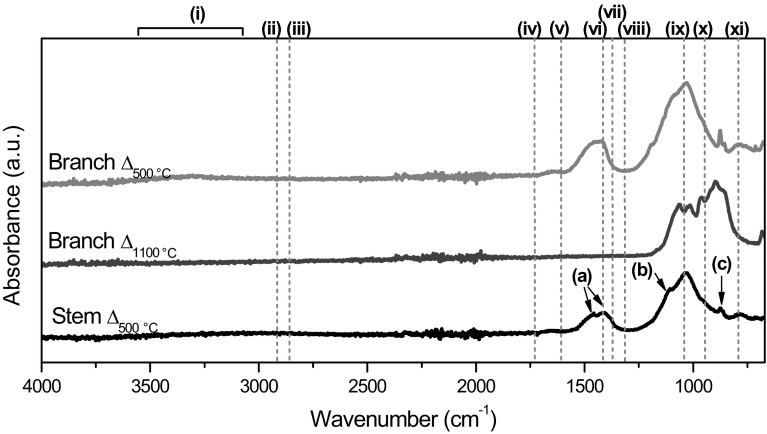
ATR spectra of thermal treated *Equisetum arvense* for the branch and the stem. No spectrum of the stem sample heated at 1100 °C is shown due to vitrification of the sample

ATR spectra of the samples after heat treatment (Fig. 8) did not show the presence of water after samples were heated at the highest temperature, 1100 °C, and only a small amount for samples heated at the lower temperature (peak i). No presence of alkyl groups (peaks ii, iii, vi, vii and viii) and/or carbonyl groups (peak iv) was detected in any of the samples. Therefore, peak vi was, in this case, no longer attributable to alkyl groups; instead, a double peak in the region of 1420–1515 cm^−1^ (peak a) probably corresponds to CO_3_^2−^ groups in silicate glasses after dissolution of CO_2_ at high temperatures and pressures relevant to the magma state [57]. These double peaks accompanied by a peak at ~872 cm^−1^ (peak c) are related to the carbonation of C–S–H when a high Ca/Si ratio is present in the sample [58, 59]. ICP-OES data showed a higher Ca/Si ratio in the samples compared with the native material, increasing from ~0.6 to ~1.1 in the branches and from ~0.1 to ~0.3 in the stems. The symmetric and antisymmetric stretching vibrations of SiO_4_ in C–S–H (peaks ix and xi, respectively) were observed in the samples heated at 500 °C. The peak at ~1115 cm^−1^ (peak b) was attributed to sulphate groups as in CaSO_4_, corroborating the crystalline phase detected by XRD in Fig. 7a [60]. At higher temperatures (1100 °C), the peaks observed in the region of 800–1100 cm^−1^ were attributed to the silicate tetrahedral vibration commonly observed for wollastonite [57].

**Fig. 9:**
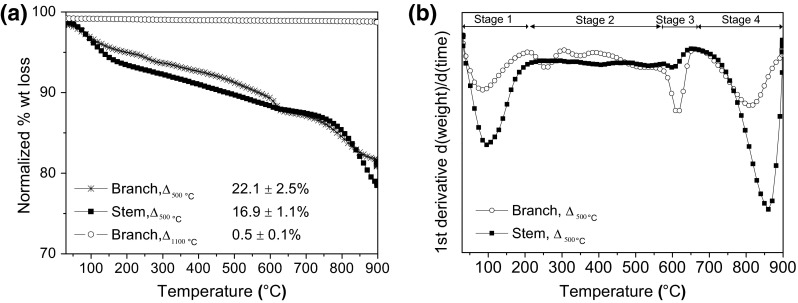
Thermal decomposition of heat-treated *Equisetum arvense* is shown as **a** TGA curves including values of total % wt loss and, **b** DTG curve for samples previously heated at 500 °C

The total % of weight loss was reduced to ~75 % when the plant material was heated at 500 °C. In the case of branches heated at 1100 °C, less than 1 % of organic material remained in the sample. Four stages of weight loss were observed for TGA analysis of samples that had previously been heated up to 500 °C (Fig. 9). In the first stage, the release of physisorbed water and/or intercalated water (*T* < 200 °C) was observed. In the second stage (200–550 °C), the weight loss observed mainly in the branches could be attributed to the release of SO_*x*_ from the crystal phase (CaSO_4_) detected in the sample (XRD data). In the third stage (550–650 °C), combustion of remaining C in the samples to CO_*x*_ was most likely. In stage four, at temperatures between 800 and 900 °C, the loss of hydroxyl groups and recrystallization of Wollastonite (CaSiO_3_) occurred [61, 62].

#### Acid washing and heat treatment under H_2_/Ar on composition

Prior to heat treatment under a reducing atmosphere, plant material was digested with HNO_3_ and H_2_SO_4_ (4:1) to remove metal ions present in the native material and isolate the biogenic silica. In this study, two batches of sample were studied: (i) complete washing after digestion and (ii) incomplete washing after digestion of the plant material.

i. Complete washing after digestion of the plant material and heat treatment under H_2_/Ar

**Fig. 10:**
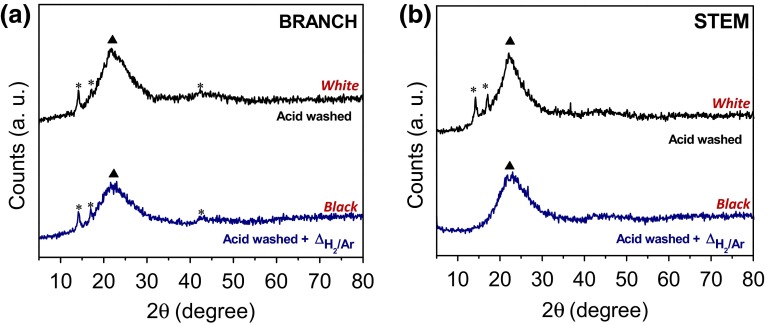
XRD diffractograms of the plant material after acid wash and then heat treatment under H_2_/Ar at 1100 °C for 5 h **a** branch and **b** stem. Crystallographic phase identified is amorphous SiO_2_ (*filled black triangle*). Peaks arising from the PP holder are labeled with an *asterisk*. Sample color after treatment is indicated in *red*

When the plant material was fully digested and carefully washed multiple times with deionized water, the ‘only’ phase detected in both, branch and stem, was amorphous silica (Fig. 10a). After heat treatment, amorphous silica was still the main phase detected (Fig. 10b) with the difference being that the samples turned black in color due to graphite generated under a reducing atmosphere. Note that, when samples are not carefully rinsed with distilled water prior to the acid treatment, the presence of α-quartz can be detected (Supplementary Figure 4). Quartz forms part of the light mineral fraction in soils together with feldspar, and nonferruginous layer silicates [63]. Therefore, contamination from the soil can give rise to confusion as to what is really present in the native plant material. From EDX analysis, all samples contained >99 % of Si and O, and other elements constituted <0.5 % of the sample (without quantification of C content). These samples contained less than <1 % of Ca and K after digestion and heat treatment and no other elements were detected by EDX and/or ICP-OES analysis. Even so, samples after heat treatment were black in color indicating the presence of C.

**Fig. 11:**
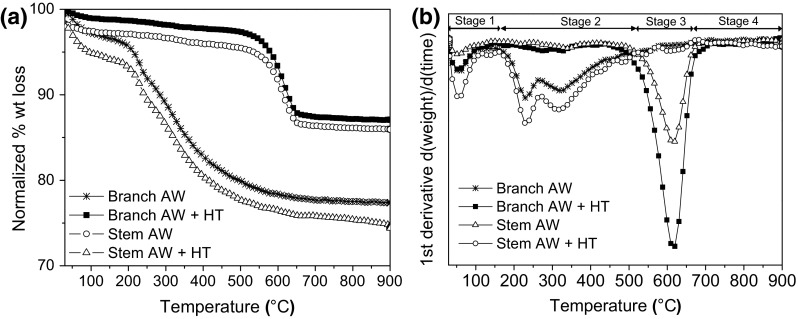
Thermal decomposition of acid washed (AW) and heat treated (HT) *Equisetum arvense* plant material is shown as **a** TGA curves and **b** DTA curves

Although amorphous silica was the only phase identified by XRD, TGA analysis (Fig. 11), showed still the presence of organic material (less than 25 %) after acid treatment with weight loss mainly in the region of 150 and 500 °C (Stage 2). The effectiveness of removal of organic matter varies in digestion period and the excess or otherwise of the acid in relation to the organic material to be removed; therefore, % of weight loss may vary between sample batches. Although heat treatment will eliminate all the remaining organic matter, it is recommended to repeat digestion if values are higher than 25 % as formation of other minerals can be generated by heating in the presence of metal ions (as previously shown). After heat treatment, the weight loss was mainly observed at temperatures between 550 and 650 °C (Stage 3) due to the combustion of C in the samples as evidenced by change of color from black to white after TGA analysis. ATR spectra of the samples (Supplementary Figure 5) only showed characteristic peaks of silica (antisymmetric and symmetric Si–O–Si stretching mode and silanol groups) and for only digested samples the presence of water, alkyl groups and silanol groups was still observed (Supplementary Figure 5).

ii. Incomplete washing after digestion of the plant material and heat treatment under H_2_/Ar

**Fig. 12:**
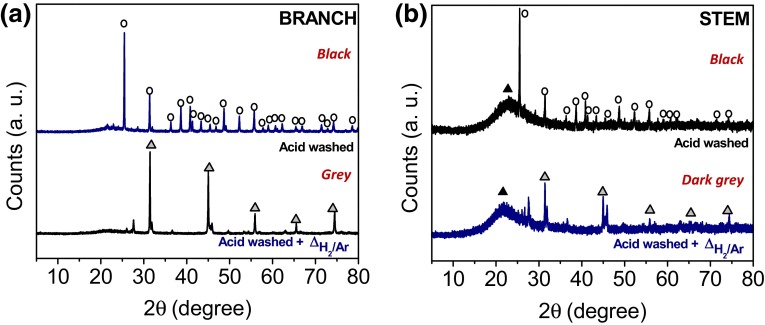
XRD diffractograms of the plant material after incomplete acid digestion and heat treated under H_2_/Ar at 1100 °C for 5 h **a** branch and **b** stem. Crystallographic phases identified are: amorphous SiO_2_ (*filled black triangle*); CaSO_4_ (*open circle*) and CaS (*filled gray triangle*). Sample color after treatment is indicated in *red*

When the samples (branch or stem) were incompletely washed following acid digestion, calcium sulphate (CaSO_4_) (JCPDS card No. 37-1496) was detected as the main crystalline phase (Fig. 12). Amorphous silica was also detected, being more apparent in the stem sample (Fig. 12b) than in the branch sample (Fig. 12a). Generally, less crystallinity was observed in the stems compared with the branches. Under a reducing atmosphere, peaks from calcium sulphate transformed to a cubic structure attributed to calcium sulphide (CaS) (JCPDS card No. 77-2011, space group *Fm*-*3* *m*). The presence of CaSO_4_ in the samples suggested that the plant material was not properly washed after acid treatment. From EDX analysis, the only elements detected were Si and O (main peaks) followed by Ca and S. ICP-OES data showed an increase of S content in the samples (compared with the native material) from ~1.6 to ~9.0 % in the branches and from ~0.4 to ~2.0 % in the stems (up to 6 times more) suggesting that the CaSO_4_ detected in the samples (without heat treatment) was possibly generated during the acid digestion. ATR spectra of the samples after acid wash and heat treatment (Supplementary Figure 4) showed the characteristic peaks of silica. In this case, digested samples also showed a peak at ~680 cm^−1^ attributed to Si–O rings besides the presence of water, alkyl groups and silanol groups (Supplementary Figure 4).

**Fig. 13:**
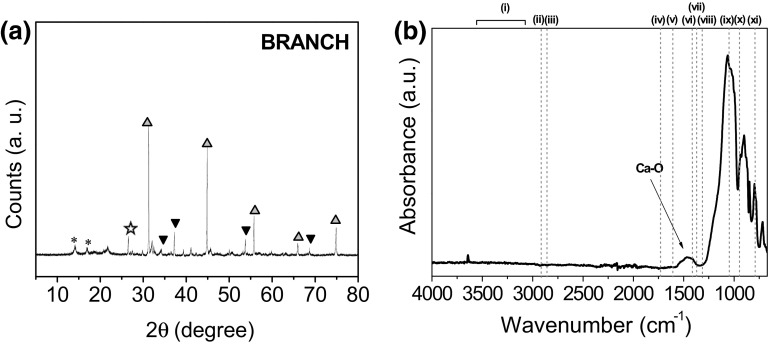
Branches treated with acetone prior to the acid wash and post-heat treatment under H_2_/Ar at 1100 °C during 5 h characterized by **a** XRD of branches treated with acetone prior to the acid wash and post-heat treatment under H_2_/Ar at 1100 °C during 5 h. Crystallographic phases identified are: CaO (*filled downward black triangle*); α-quartz (*open star*); and CaS (*filled gray triangle*) where peaks arising from the PP holder are labeled with an *asterisk*, and **b** ATR spectrum

During this set of experiments, branches were also treated with acetone prior to the acid wash and post-heat treatment under H_2_/Ar in order to extract the chlorophyll present in the plant. In this case, samples collected at the end of all treatments were no longer black but yellowish. XRD analysis, showed the presence of CaS previously observed for the non-acetone treated samples but also calcium oxide (CaO) with cubic structure (JCPDS card No. 70-5490, space group *Fm*-*3* *m*) (Fig. 13a). The formation of CaO can occur by the reaction of CaS with an excess of unreduced CaSO_4_ [64]. The presence of α-Quartz most probably arose from soil contamination. In ATR spectra (Fig. 13b), a broad peak arising at ~1463 cm^−1^ was indicative of a CaO phase in the structure [65]. The peak detected at ~680 cm^−1^ could be attributed to Si–O rings; however, the presence of a peak at ~902 cm^−1^ and the irregular shape of the peaks, showed similarities with the CaSiO_3_ (Wollastonite) previously found in the branches heat treated at 1100 °C. If present, this was clearly a minor component of the sample as the mineral was not detected by XRD.

### Luminescence behavior

The samples that showed luminescence, Fig. 1, either contained CaS generated by the chemical/thermal treatment process or quartz as a result of soil contamination that had not been removed prior to sample treatment and cannot completely be excluded as a possibility. For the latter, luminescence properties have been related to the presence of defects in its crystal lattice [66] which can be caused by the presence of other elements, in this case, by the presence of ions from CaS and/or CaO. For CaS, a study carried out by W. Lehman showed 31 different possible co-activators of CaS phosphors (i.e. Si, K, Na, P or Cl) each producing its own emission color. Interestingly, blue-green emission (as in this study) was observed with addition of CaO to CaS [67].

## Conclusions

*Equisetum arvense* contains silica and other elements including Ca, K, S, Na, Mg, Cl and P with varying distribution patterns across the plant. Silica was essentially detected on the spotted surfaces which generally contained a stomatal shape, whereas the multi-laminate layers showed a higher amount of K and Ca as well as lower amounts of S, Na, Mg, Cl and P. Higher levels of calcium were found in the branches, whereas K was found at higher levels in the stems. A higher crystallinity was observed in samples obtained from branches compared to the stems, with the latter showing higher silicon (ICP-OES) levels as amorphous silica (XRD). For this plant, solation of ‘pure’ biogenic silica was only achieved by pre-treatment of samples with concentrated acids. When heat treatment under air was applied to the plant material, crystalline phases other than amorphous silica were isolated due to the presence of metal ions in the plant material such as Ca and K, producing crystalline phases of these elements with or without combination with silicon. As K was more likely to be found in the stems, heat treatment gave rise to the formation of KCl, whereas branches, with generally a higher fraction of Ca, formed calcium sulphate and/or calcium silicates (monohydrate or wollastonite). When stems were subjected to a high temperatures (>1000 °C) vitrification of the sample was observed. The presence of α-quartz peaks in the samples was shown to arise from soil contamination. When samples were not efficiently washed after digestion, the presence of sulphates could be detected in the heated samples, which subsequently formed sulphides by heat treatment under reducing conditions. Luminescence was observed for a sample which contained CaS and CaO or α-quartz.

## Electronic supplementary material

Supplementary material 1 (PDF 657 kb)
