# Supplementary material for: Isolation of a wide range of minerals from a thermally treated plant: Equisetum arvense, a Mare’s tale
Source: J Biol Inorg Chem. 2016 Jan 13;21:101–12. doi: 10.1007/s00775-015-1320-0 (PMC4771831; doi:10.1007/s00775-015-1320-0)

Electronic Supplementary Material

Isolation of a wide range of minerals from a thermally treated plant: *Equisetum arvense*

Anna Sola-Rabada[1] · Julia Rinck[2,3] · David J. Belton[1] · Annie K. Powell[2] · Carole C. Perry[1]✉

[1]Interdisciplinary Biomedical Research Centre, Nottingham Trent University, Clifton Lane, Nottingham, UK, NG11 8NS, [2]Karlsruhe Institute of Technology, Institute of Inorganic Chemistry, Engesserstrasse 15, 76131 Karlsruhe, Germany and [3]Karlsruhe Institute of Technology DFG-Center for Functional Nanostructures (CFN), Wolfgang-Gaede-Straße 1a, 76131 Karlsruhe, Germany

TABLE OF CONTENTS:

Figure S1: SEM images with corresponding mapping for structures in the native plant material.

Figure S2: From TGA data, % of weight loss in the native material (Branch and Stem).

Figure S3: XRD diffractogram of the sample vitrified sample (stem from *Equisetum Arvense* plant) after heat treatment at 1100 ºC under air.

Figure S4: XRD of branches with or without ddH_2O wash prior to the acid treatment.

Figure S5: ATR spectra of *Equisetum Arvense* after (a) complete acid digestion and thermal treatment and (b) incomplete acid washed/digestion and thermal treatment.

Figure S1

SEM images with corresponding mapping for structures in the native plant material.

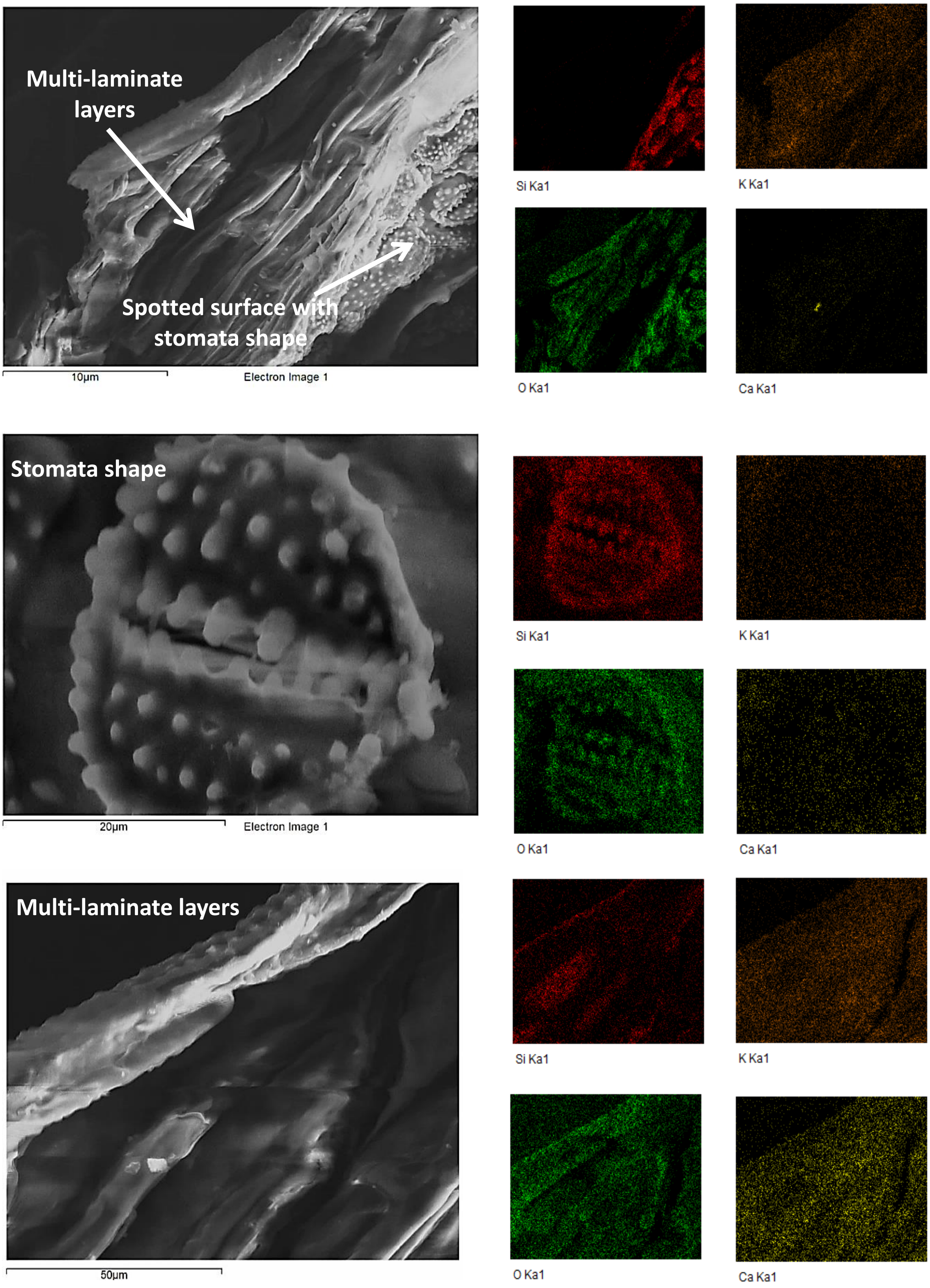

Figure S2

From TGA data, % of weight loss in the native material (Branch and Stem):

- Stage 1: physisorbed water or volatiles oils present in the native material (T < 120 °C)
- Stage 2: degradation of hemicellulose and cellulose (T ~290 ºC)
- Stage 3: degradation of lignin (T = 400-800 ºC)
- Stage 4: dehydroxylation of silanols groups (T = 800-900 ºC)

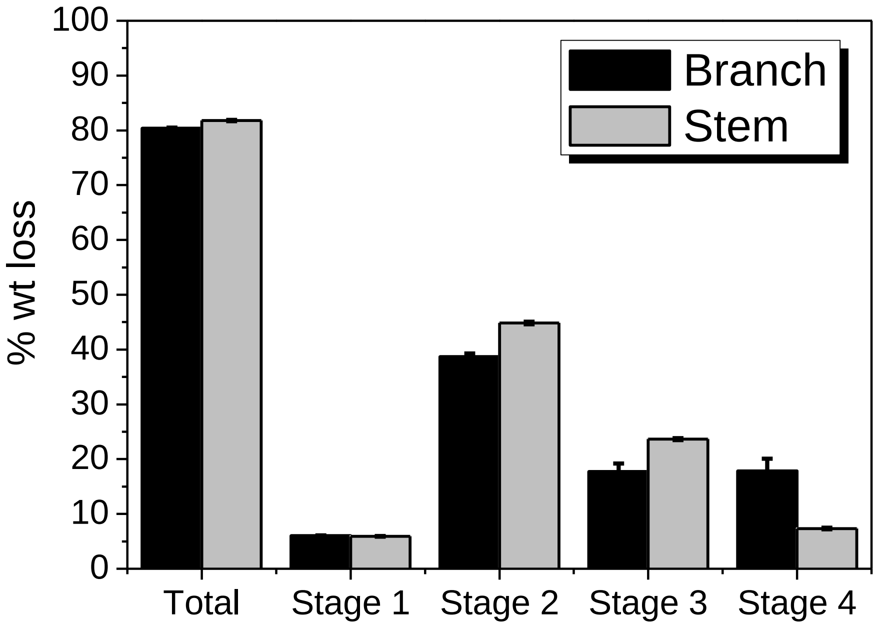

Figure S3

XRD diffractogram of the sample vitrified sample (stem from *Equisetum Arvense* plant) after heat treatment at 1100 ºC under air. Peak position and *d*-spacing values arising from vitrified phase are given.

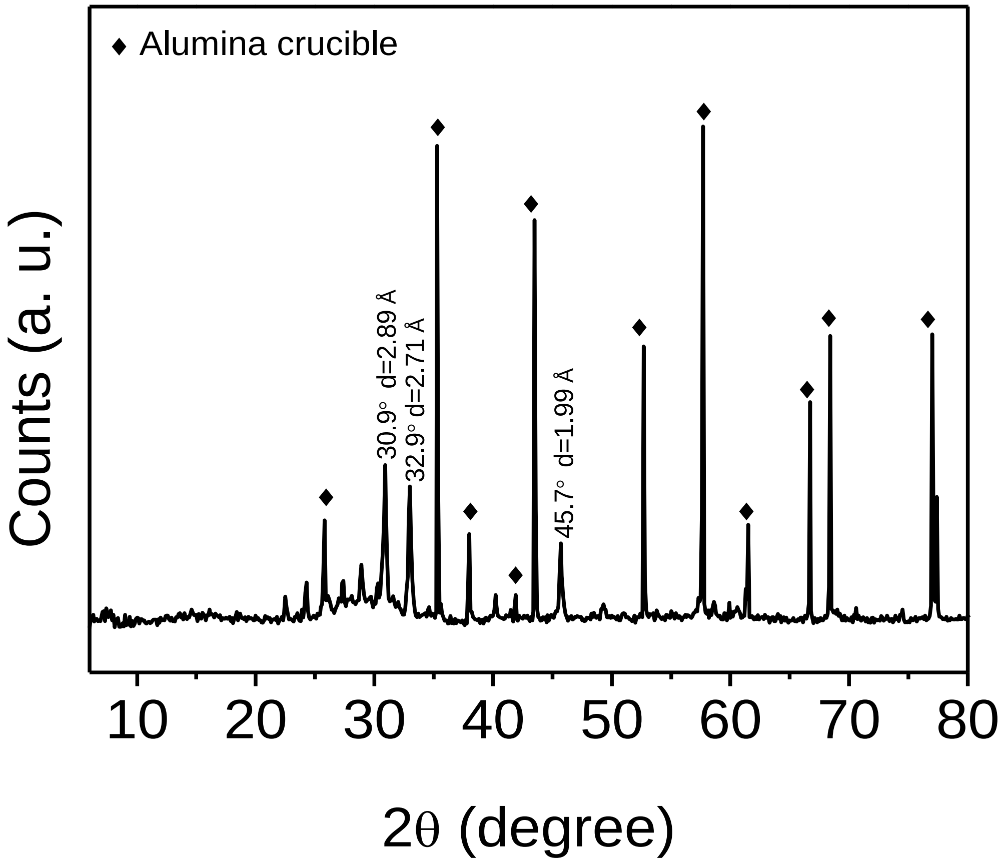

Figure S4

XRD of branches with or without ddH_2O wash prior to the acid treatment. Branches showing quartz peak (☆) when samples have not been properly rinsed with ddH_2O prior to the acid treatment. Amorphous silica is marked with ▲.

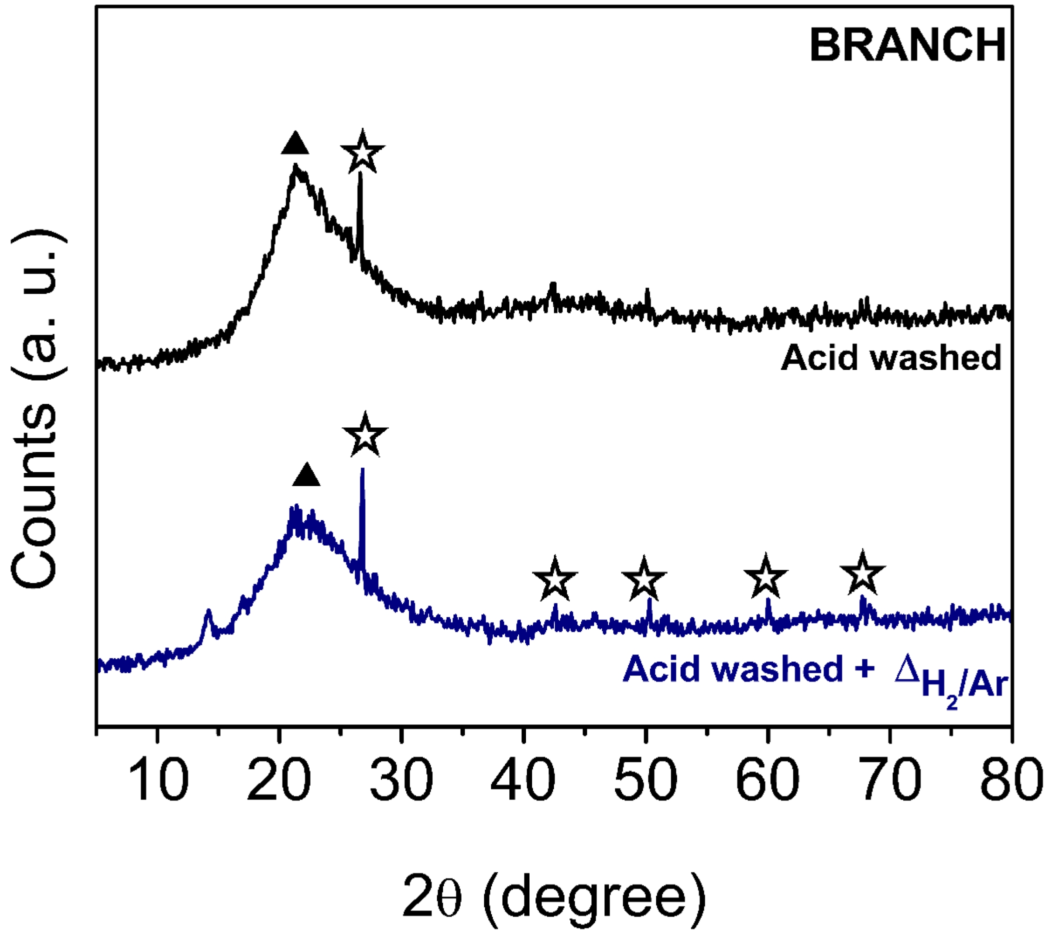

Figure S5

ATR spectra of *Equisetum Arvense* after:

(a) complete acid digestion and thermal treatment

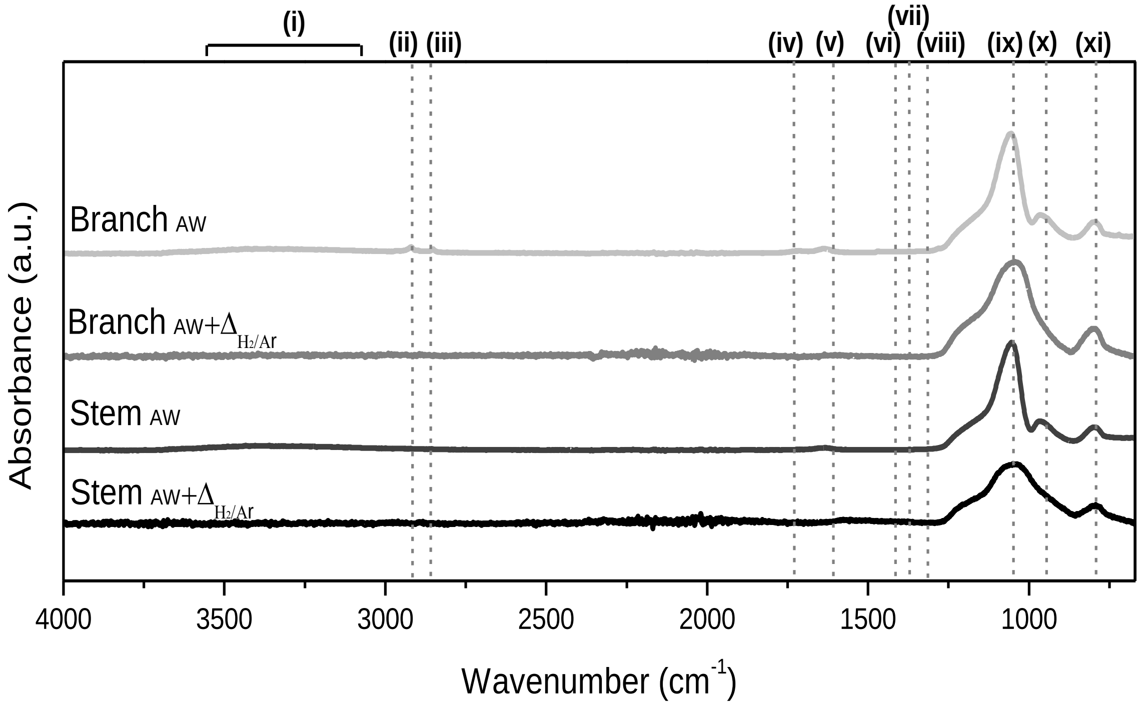

(b) incomplete acid washed/digestion and thermal treatment

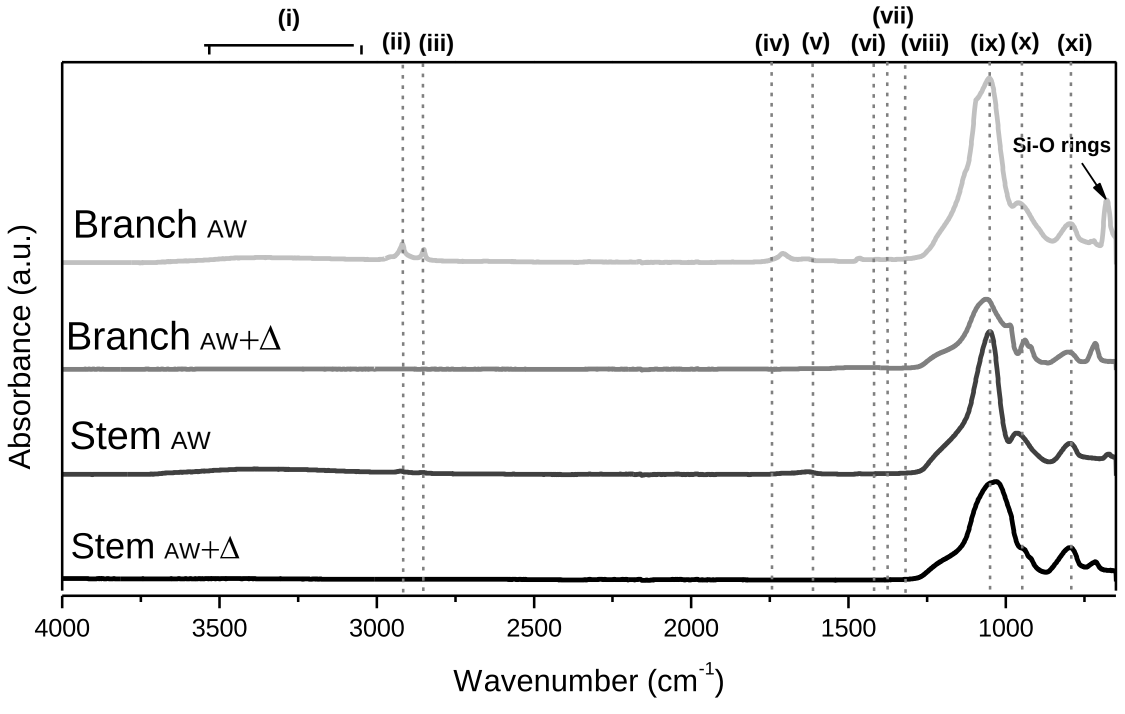

Supplement: Supplementary file 1 — Supplementary material 1 (PDF 657 kb) [file 775_2015_1320_MOESM1_ESM.pdf]
